# Genetic and Sex Associations with Earlier Estimated Onset of Amyloid Positivity from over 4000 Harmonized Positron Emission Tomography Images

**DOI:** 10.1002/alz.092852

**Published:** 2025-01-09

**Authors:** Tonnar Castellano, Ting Chen Wang, Emma Nolan, Derek B. Archer, Karly Cody, Theresa M. Harrison, Yiyang Wu, Alaina Durant, Vaibhav A Janve, Corinne D. Engelman, William J. Jagust, Marilyn S. Albert, Sterling C. Johnson, Susan M. Resnick, Reisa A Sperling, Murat Bilgel, Andrew J. Saykin, Badri N Vardarajan, Richard Mayeux, Tobey J. Betthauser, Logan C. Dumitrescu, Elizabeth Mormino, Timothy J. Hohman, Mary Ellen I. Koran

**Affiliations:** ^1^ Vanderbilt Memory and Alzheimer’s Center, Institute for Medicine and Public Health, Vanderbilt University Medical Center, Nashville, TN USA; ^2^ Vanderbilt Genetics Institute, Institute for Medicine and Public Health Vanderbilt University Medical Center, Nashville, TN USA; ^3^ Vanderbilt University, Nashville, TN USA; ^4^ Vanderbilt Memory and Alzheimer’s Center, Vanderbilt University Medical Center, Nashville, TN USA; ^5^ Stanford University, Stanford, CA USA; ^6^ University of California, Berkeley, Berkeley, CA USA; ^7^ University of Wisconsin‐Madison, School of Medicine and Public Health, Madison, WI USA; ^8^ Johns Hopkins University School of Medicine, Baltimore, MD USA; ^9^ Alzheimer’s Disease Research Center, University of Wisconsin School of Medicine and Public Health, Madison, WI USA; ^10^ National Institute on Aging, National Institutes of Health, Baltimore, MD USA; ^11^ Massachusetts General Hospital, Boston, MA USA; ^12^ Center for Alzheimer Research and Treatment, Department of Neurology, Brigham and Women’s Hospital, Boston, MA USA; ^13^ Indiana University School of Medicine, Indianapolis, IN USA; ^14^ The New York Presbyterian Hospital, New York, NY USA; ^15^ Taub Institute for Research on Alzheimer’s Disease and the Aging Brain, Columbia University, New York, NY USA; ^16^ The Institute for Genomic Medicine, Columbia University Medical Center, New York, NY USA; ^17^ Columbia University Medical Center, New York, NY USA; ^18^ Taub Institute for Research on Alzheimer’s Disease and the Aging Brain, Columbia University Medical Center, New York, NY USA; ^19^ University of Wisconsin School of Medicine and Public Health, Madison, WI USA; ^20^ Alzheimer’s Disease Research Center, University of Wisconsin‐Madison School of Medicine and Public Health, Madison, WI USA; ^21^ Stanford University, Palo Alto, CA USA

## Abstract

**Background:**

New techniques have been developed to estimate the age when someone converted to amyloid positivity (EAOA) from PET, oftentimes offering information Aβout a participant decades before they joined a research study. EAOA is variable across populations but we do not know the causes for these differences. This study aims to validate *APOE* associations with EAOA and explore genetic and sex‐based factors with EAOA.

**Methods:**

Data from six cohorts were analyzed. Our analysis included 4220 non‐Hispanic white people (57.6% women; 86.7% cognitively unimpaired at baseline scan). Amyloid PET data were harmonized using gaussian mixture models. EAOA was calculated using the sampled iterative local approximation (SILA) algorithm. Sex differences in EAOA were compared using t‐tests amongst amyloid positive individuals. A genome‐wide association study of EAOA was performed. Gene analyses were conducted using MAGMA.

**Results:**

Average EAOA was 81.1 years across all individuals regardless of amyloid status. *APOE* e2 homozygotes had slightly later EAOA than e3/e3 homozygotes. *APOE* e4 homozygotes converted to amyloid positivity 8.2 years before e3/e4 heterozygotes and over two decades earlier than e3 homozygotes. *APOE* e2/e4 converted to positivity roughly three years later than e3/e4 and nearly ten years earlier than e3 homozygotes. *APOE* genotype differences in EAOA described were statistically significant (p < .01). There were significant sex differences between men and women when examining amyloid positivity. Men converted to amyloid positivity over 2 years later than women (65.3 vs 63.2 years, p=3.23x10‐5). The rs12981369 polymorphism in *ABCA7* was associated with EAOA (β = 2.14, p=9.27×10−9). Brain eQTL databases indicate associations between rs12981369 and gene expression of *ABCA7*. Gene‐level analyses revealed significant associations for *ABCA7, HMHA1*, and *KIF13B*.

**Conclusion:**

This study further describes the role of *APOE* and reveals roles for *ABCA7* and *KIF13B* on amyloid onset. We identified a novel variant on chromosome 19 correlating with later amyloid onset conversion and highlight important differences between sexes. These findings highlight EAOA as a powerful endophenotype of AD and offer insights into potential drug‐targetable mechanisms for early AD intervention.

